# The path to fusion power^†^

**DOI:** 10.1098/rsta.2009.0216

**Published:** 2010-03-13

**Authors:** Chris Llewellyn Smith, Steve Cowley

**Affiliations:** Euratom/CCFE Fusion Association, Culham Science Centre, Abingdon OX14 3DB, UK

**Keywords:** energy, fusion, power

## Abstract

The promise, status and challenges of developing fusion power are outlined. The key physics and engineering principles are described and recent progress quantified. As the successful demonstration of 16 MW of fusion in 1997 in the Joint European Torus showed, fusion works. The central issue is therefore to make it work reliably and economically on the scale of a power station. We argue that to meet this challenge in 30 years we must follow the aggressive programme known as the ‘Fast Track to Fusion’. This programme is described in some detail.

## 1. Introduction

Global energy use is expected to increase 45 per cent by 2030 (World Energy Outlook 2008). Meeting this demand will be extremely difficult; meeting it in an environmentally responsible manner will be an enormous challenge.

Currently, 80 per cent of the world’s primary energy is generated by burning fossil fuels—a resource that is rapidly dwindling. Consider coal reserves. It is often said that there is enough coal for some 200 years—but that assumes current use. Continuation of the current 4.5 per cent per annum growth in coal use reduces 200 years to 50. And it is well established that burning fossil fuels has adverse effects on climate and the environment (Intergovernmental Panel on Climate Change 2007).

All experts agree that a portfolio approach is needed to decrease dependence on fossil fuels and to meet increased world energy demand—there is no single ‘solution’. Improved efficiency will be vital, and needs to be encouraged by fiscal measures and regulations. But, even if efficiency can reduce demand in the developed world, it cannot provide the growth in total energy use that is needed to deliver a decent standard of living in the developing world, where 1.5 billion people still lack electricity. Renewables should be deployed to the largest extent reasonably possible. But, leaving aside solar power for the moment, it is almost impossible to imagine renewables providing more than half of the energy currently supplied by fossil fuels.

If we ask what is needed to take over the role of fossil fuels, there are only three candidates with sufficient resource for the long term—solar, nuclear fission with uranium or thorium breeders and nuclear fusion. All three require substantial research and development before they are ready to be deployed on a large scale. Research on all three options should be a priority. In this paper, we focus on the promise, status and challenges of developing fusion as a power source.

## 2. The promise of fusion

There are four key attributes that make fusion a desirable technology for power production.

### (a) Essentially unlimited fuel supply

The fuels for the easiest fusion reaction are deuterium and tritium which can be ‘bred’ from lithium. There is enough deuterium in water for billions of years of world energy consumption and enough easily mined lithium for several hundreds of years. One kilogram of natural lithium yields about 3×10^13^ J or equivalent to 9×10^5^ l of petrol. When lithium becomes scarce on land, it could be extracted from water, which contains enough to power the world for a few million years (Fasel & Tran 2005).

### (b) Safe operation

The fusion reacting ‘plasma’ in a fusion power plant would contain insufficient fuel to produce a dangerous runaway reaction or cause serious damage to the plant. Should the cooling circuit fail completely, radioactivity in the walls would continue to generate heat, but the temperature would peak well below the temperature at which the structure could melt. Fusion power plant designs minimize the inventory of radioactive isotopes (such as tritium) so that the worst imaginable accidents or incidents (such as earthquakes or aircraft crashes) would not require evacuation of the neighbouring population.

### (c) Low land use

A fusion power plant would occupy roughly the same space as a fission or fossil fuel plant. Because of their intrinsic safety fusion plants could be placed on sites relatively close to population centres and other major consumers of energy.

### (d) Minimal waste and low environmental impact

Although the by-products of fusion (helium and neutrons) are not radioactive, the structure will become activated when struck by the fusion neutrons. With appropriately chosen structural materials, however, the radioactive products will decay with half-lives of the order of 10 years, and all the components could be recycled within 100 years.

Clearly, fusion’s promise is huge. The fact that a tiny amount of lithium can produce so much electricity, without any production of CO_2_ or air pollution, is sufficient reason to develop fusion urgently even if success is not 100 per cent certain. There are no known or expected obstacles but there are certainly challenges. The most obvious challenge—holding fusion fuel at temperatures 10 times hotter than the centre of the sun—has in fact been surmounted. Experiments at the Joint European Torus (JET) in the UK regularly achieve such conditions. Indeed, JET has produced 16 MW of fusion power (Keilhacker *et al.* 1999), and the International Tokamak Experimental Reactor (ITER; the international fusion experiment being constructed in France) is expected to produce 500 MW for hundreds of seconds at a time.

So fusion works. The big question is: when will it be made to work reliably and economically on the scale of a power station? Before attempting to answer, we consider the following questions. What is fusion? What will a fusion power station look like? What has been achieved? What are the outstanding science and technological challenges? We will then argue that attaining the first fusion electricity in about 30 years (the EU goal) is challenging but possible. Every attempt should be made to reduce the timescale further.

## 3. What is fusion?

The fusion reaction of primary interest as a source of power on Earth involves two isotopes of hydrogen (deuterium, D=^2^H, and tritium, T=^3^H) fusing to form helium and a neutron,3.1
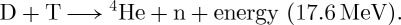

Energy is liberated because helium-4 is very tightly bound: it takes the form of kinetic energy, shared 14.1 MeV/3.5 MeV between the neutron and the helium-4 nucleus. This reaction has a cross section about a hundred times larger than any other fusion reaction at energies of interest. Tritium is unstable with an approximately 12 year half-life and thus it has to be bred from lithium using the neutron from the fusion reaction (3.1) and the reactions
3.2
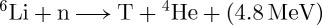
and
3.3
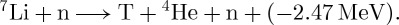

Thus, the fuels are deuterium and lithium. The reaction (3.2) dominates (3.3) in many designs even though ^6^Li is only approximately 7.5 per cent of natural lithium. One kilogram of natural lithium yields about 3×10^13^ J or equivalent to 9×10^5^ l of petrol. To initiate the fusion reaction (3.1), the charged deuterium and tritium nuclei must get close enough for the strong nuclear force to act and bind the helium nucleus together. To get this close, they must approach each other fast enough to overcome their mutual electrical repulsion. Thus, a gas of deuterium and tritium must be heated to over 100 million degrees Celsius (10 KeV)—10 times hotter than the core of the sun—for sufficient nuclei to have the required kinetic energy to fuse. This gas is in fact a plasma since, above a few thousands degrees, inter-atomic collisions knock the electrons out of the atoms to form a mixture of separated nuclei and electrons.

In the temperature range 100–200 million degrees Celsius the fusion power per unit volume (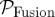) from a D–T plasma is given roughly by3.4
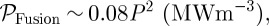

where *P* is the plasma pressure in atmospheres. It is immediately clear from equation (3.4) that the D–T reaction produces commercially viable power densities (megawatts per metre cubed) at practically sustainable pressures (a few atmospheres). The remarkably large D–T cross section puts fusion power within reach!

Many conceptual schemes for using reactions (3.1)–(3.3) to generate power have been proposed. However, the tokamak (see below) is the only device to have produced significant fusion (on JET and the Tokamak Fusion Test Reactor (TFTR) in the USA). Moreover, careful studies of power plants based on the tokamak show that they provide a promising route to commercial fusion power (see below and EU Studies (2004)). In this paper, we focus mainly on the tokamak approach. We will not make any remarks about the inertial approach to fusion since it is, at present, a generation behind magnetic fusion.

## 4. What will a fusion power station look like?

**Figure 1.**
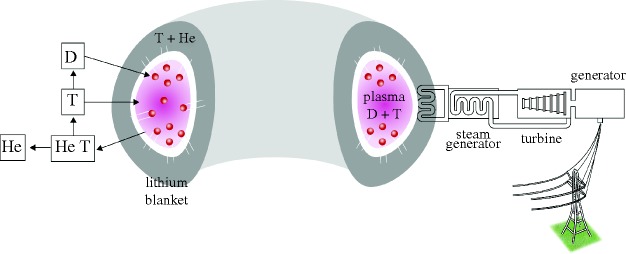
A conceptual tokamak fusion plant (not to scale) showing the three parts: a fusion burning D–T plasma; a lithium ‘blanket’; and a turbine-driven generator delivering electricity to the grid.

 figure 1 shows the conceptual layout of a tokamak fusion power station. Fusion power stations will be similar to existing thermal power stations, but with a different furnace and fuel. We describe a typical design (roughly model B of the fusion power plant designs (EU Studies 2004)) with approximate numbers to set the scale.

At the centre is a fusion burning D–T plasma with a volume of approximately 2000 m^3^ confined in a ‘toroidal’ (doughnut shaped) chamber by a strong magnetic field (approx. 5–6 T). D and T are fed into the core and heated to over 100 million degrees Celsius—typically there is of the order of a gram of fuel in the device at any moment. The D–T reaction (equation (3.1)) produces about 3.6 GW of fusion power. The helium nuclei (alpha particles) from the fusion reactions are confined by the magnetic field. They are slowed down by collisions on the deuterium and tritium thereby depositing about 700 MW of the fusion power in the plasma. This heating sustains the plasma temperature against leakage of energy across the magnetic field.

The neutrons produced by the fusion reaction (3.1) escape the magnetic bottle and penetrate the surrounding structure, known as the blanket, which will be about 1 m thick. In the blanket, the neutrons collide with lithium and breed tritium through the reactions in equations (3.2) and (3.3). Most of the tritium is produced by the reaction with ^6^Li (equation (3.2)). Since natural lithium is about 7.5 per cent ^6^Li, some blanket designs envisage enhancing the ^6^Li. There are also various competing reaction channels, which do not produce tritium, but in many cases produce additional neutrons that in turn can produce tritium. The production of additional neutrons can be enhanced, e.g. by adding beryllium or lead. The result is that, on paper at least, it is possible to design fusion reactors that would produce enough tritium for their own use, plus a small surplus to start up new plants. For safety reasons it is desirable to keep the tritium inventory low. Thus, it is important to remove the tritium from the blanket regularly.

The neutrons will also slow down and deposit their energy (4/5ths of the fusion power—about 2.8 GW) in the blanket. The blanket will heat up to around 400^°^C in the so-called ‘near-term’ power plants that would use relatively ordinary materials. In advanced models that use materials such as silicon carbide it could conceivably reach 1000^°^C. The heat will be extracted through a primary cooling circuit, which could contain water or helium, which in turn will heat water in a secondary circuit that will provide the steam/gas to drive turbines. These turbines generate about 1.5 GW of electricity (enough for more than a million people) in the conventional way. For economic viability, the blanket must operate robustly for many years. Because this component is both critical and unique to fusion it will contain much of the intellectual property in fusion. A 1.5 GW plant would use about 10–20 kg of natural lithium and 0.6 kg of deuterium per day and the electricity is estimated to cost in the range 5–9 Eurocents per kW h.

## 5. Status of fusion research: tokamaks

**Figure 2.**
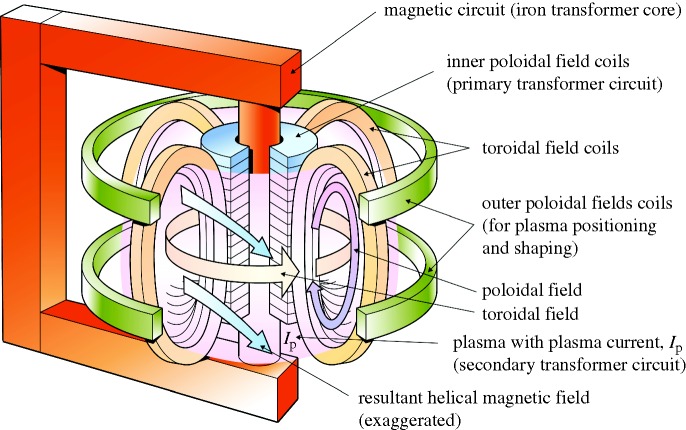
In a tokamak, the fusion fuel is held in a toroidal chamber surrounded by magnets. A current is induced in the fuel by transformer action and, together with the toroidal field coils, produces a helical magnetic structure that holds the hot fuel away from the wall.

The leading magnetic configuration for confining fusion plasmas is called a tokamak (a contraction of a Russian phrase meaning toroidal chamber with a magnetic coil). The basic layout of a tokamak is shown in figure 2. In this section we outline the basic operation of a tokamak. A fusion plasma configuration is made in the tokamak via the following steps:

— A small amount of gas (hydrogen or deuterium in most experiments; deuterium and tritium in some experiments at JET and in an actual fusion reactor) is injected into the toroidal (doughnut shaped) vacuum chamber after the magnetic field coils have been switched on.
— A rising current is raised in the inner poloidal field coils (see figure 2) that drives an electric current (approx. 5 MA in JET and approx. 15 MA in ITER) through the gas, via transformer action.
— The electric current heats the gas, and turns it into a plasma. It also produces a magnetic field which, combined with the magnetic field produced by the external coils, generates a helical field that is necessary to ‘confine’ the plasma, i.e. hold it away from the walls and provide very good thermal insulation.
— The electrical resistivity of a plasma drops rapidly with temperature and the current induced by transformer action can heat the plasma to only about one-third of the temperature needed for copious fusion to occur. Additional heating power (of many megawatts) must then be supplied by microwaves or beams.
— The transformer can sustain the plasma current only while the current is rising in the inner poloidal field—thereafter the current must be driven by the microwaves and or beams of energetic neutral atoms which transfer energy to the plasma through collisions.
— When the plasma reaches temperatures of about 100 million degrees Celsius the D–T fusion reactions begin. The helium (alpha particles) nuclei from the fusion reaction then heat the plasma.

In addition to heating and current drive systems, experimental tokamaks are equipped with ‘diagnostic’ devices that measure the magnetic field, electron and ion temperatures and densities, the plasma pressure, position and shape, neutron and photon production, impurities, etc., and monitor the development of instabilities.

## 6. Performance measures—what plasma performance has been achieved?

In this section we define the three key plasma performance measures—the energy confinement time, *τ*_E_; the usage of the magnetic field, *β*; and the current drive efficiency—and review the performance that has been achieved. It is necessarily technical and can be omitted by the reader focused on fusion policy.

The power balance in the plasma is critical to fusion performance. One-fifth of the fusion power is produced as kinetic energy of helium nuclei (alpha particles). The alpha particles heat the plasma. Let us denote the external heating power density from microwaves or beams as 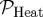. The heat losses are almost entirely from turbulent transport of heat across the magnetic field—the leakage. We parameterize these losses by the ‘energy confinement time’ (*τ*_E_) defined by
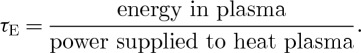

**Figure 3.**
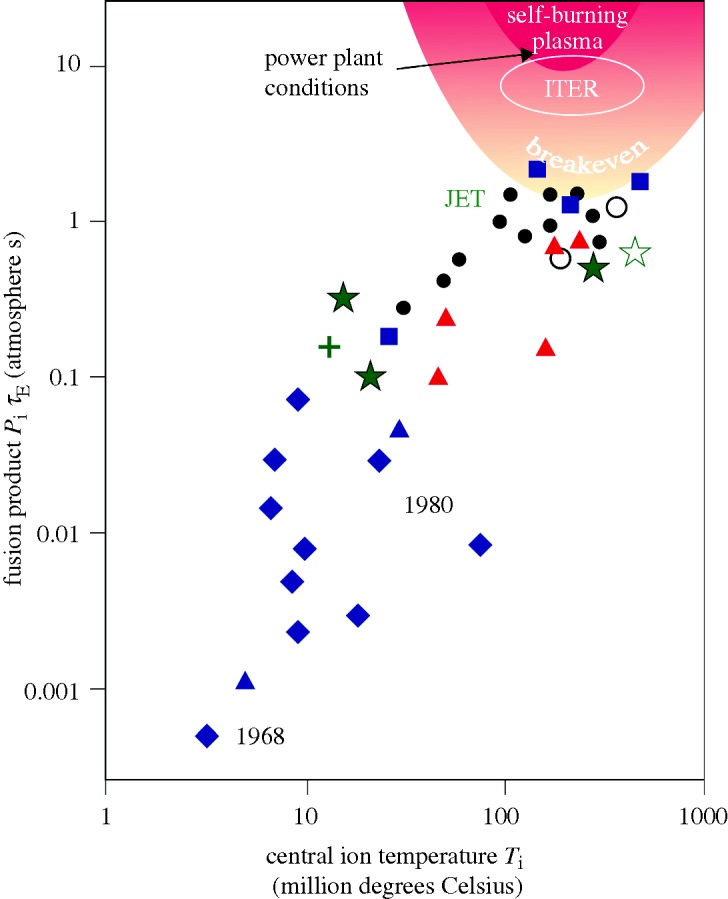
Selected results from different tokamaks demonstrate substantial progress over recent decades from the low temperature, low energy gain points at the bottom left. Temperatures above 100 M^°^C are now routinely achieved and an energy gain of around one has been reached. A power plant needs an energy gain (*Q*) above 10, and this should be achieved in ITER.

*τ*_E_ measures how well the magnetic field insulates the plasma. It is obvious that the larger *τ*_E_, the more effective a fusion reactor will be as a net source of power. In steady state the power balance per unit volume is6.1
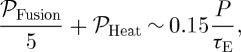

where, as before, we express power density in MW m^−3^ and pressure in atmospheres. The energy gain is defined by 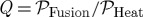. At sufficient pressure the plasma is entirely self-heated and 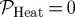 and 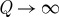; this is termed ignition. High gain is essential for commercial fusion as supplying 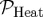 reduces the net output and complicates reactor design. The ‘fusion product’, *Pτ*_E_, and the temperature, *T*, determine the energy gain of the fusion device. In the temperature range 100–200 million degrees Celsius (where equation (3.4) applies) ignition occurs when *Pτ*_E_>20 (*P* in atmospheres and *τ*_E_ in seconds). The ‘fusion performance plot’ (figure 3) of *P*_i_*τ*_E_ versus *T* (*P*_i_=*P*/2 is the ion pressure) shows the breakeven point (*Q*=1) and data points from different tokamaks, and indicates the substantial progress towards power station conditions that has been achieved in recent decades.

Much of this progress in tokamak performance over 40 years has been achieved by suppressing the turbulent convection of heat and thereby increasing *τ*_E_. The turbulent ‘eddies’ are a few ion larmor radii (*ρ*_*i*_) across (typically a centimetre) and they are highly elongated along the magnetic field lines (typically with a length the size of the tokamak, *L*(m)). These eddies turn over in a time *L*/*v*_i_, where *v*_i_ is a typical ion velocity. The theory of this turbulence is complicated and just beginning to be understood. Progress has been greatly helped by high performance computations. However, the rough scaling of the confinement can be estimated by considering the turbulent motion to be a random walk of step size *ρ*_i_ and correlation time *L*/*v*_i_—this yields 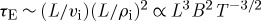. The experimental/empirical confinement scaling differs a little from this simple estimate. Nonetheless, strong dependence on size and magnetic field is observed.

In 1982 the serendipitous discovery (in a fusion experiment at Garching in Germany (Wagner *et al.* 1982)) of a ‘high-confinement’ plasma mode (H mode) revealed the important role played by plasma rotation. In these plasmas a narrow shear layer develops at the edge of the plasma—in this layer the turbulence is suppressed by shear in the plasma rotation. Theory confirms that when the shearing rate is faster than the eddy turnover the sheared rotation rips apart the turbulent eddies and suppresses heat transport. Just how the plasma spontaneously creates shear layers is not fully understood. Nonetheless, ITER will rely on the H mode for its highest performance. If turbulence could be suppressed throughout the plasma, tokamak reactors could be made considerably smaller (and cheaper) than currently predicted.

The magnetic field provides a force (**J**×**B**) to contain the plasma pressure—roughly speaking this is a magnetic pressure. In atmospheres the magnetic pressure is6.2
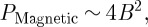

where *B* is measured in tesla and *P*_Magnetic_ is in atmospheres. JET operates at up to 4 T and ITER will operate at up to 5.2 T. The cost of the coils can be over 30 per cent of the cost of a fusion reactor. A measure of the efficiency of confinement is the ratio between plasma and magnetic pressure6.3
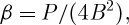

where, as before, *P* is in atmospheres and *B* in tesla. Since the fusion power is proportional to *β*^2^*B*^4^ (see equation (3.4)) achieving high *β* is critical to large fusion power and thereby commercial success. However, when *β* exceeds a critical value the plasma is unstable and disrupts rapidly—these instabilities are understood and accurately predicted by theory. Shaping the magnetic configuration and rotating the plasma have increased the achievable *β*. The recently developed spherical tokamaks (where the ratio of minor to major radius is approx. 1) achieve remarkable *β* values. For example, JET achieves *β* values of a few percent; MAST, the spherical tokamak at Culham, can achieve *β*’s of more than 30 per cent; ITER will operate at *β* of 4–5%.

When the plasma is burning at high gain the energy needed to sustain plasma is negligible. However, for continuous operation the current must be sustained against resistive decay. For efficient fusion it is essential that the external power (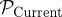) needed to drive the current be small compared with the fusion power. Culham researchers (Bickerton *et al*. 1971) showed that if the plasma pressure (*β*) is sufficiently high the current could in principle be sustained by a collisional effect—this they termed ‘bootstrap’ current. The bootstrap current was first measured in a tokamak in 1988 (Zarnstorff *et al.* 1988). It is important to maximize the bootstrap current since it requires no external power. Current has been sustained indefinitely in several experiments with a combination of bootstrap and radio frequency or beam current drive (Becoulet & Hoang 2008). The fraction of the current driven by the bootstrap often exceeds 50 per cent and can reach 100 per cent (Becoulet & Hoang 2008). ITER is expected to approach steady state with a partially bootstrap-driven current in its so-called hybrid modes.

We can be rather confident that ITER (see below) will reach the burning plasma region indicated in figure 3. The extrapolation from JET to ITER is generally modest: the ITER operating temperature and *β* are regularly achieved in JET. However, the confinement time must increase from less than 1 s to 3–4 s. ITER is twice as big as JET in every dimension and the magnetic field is increased from approximately 3–4 T to 5 T. The semi-empirical scaling laws that interpolate rather accurately between results from machines with very different sizes, magnetic fields and plasma currents yield an ITER confinement time of 4 s—slightly less than the random walk result (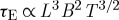). Negative developments are of course not excluded in the future. There could be new instabilities in the burning plasmas that will be studied, for the first time, at ITER. An obvious candidate is the excitation of Alfvén waves triggered by slowing alpha particles when they pass through the Alfvén velocity. But theoretical and experimental simulations suggest that this is very unlikely.

Tokamaks promise to scale to commercial reactors. Nonetheless, two alternative magnetic configurations are being pursued: the spherical tokamak and the stellarator. The spherical tokamak has two key advantages over the conventional tokamak: high *β* (see above) and compactness. This makes it a promising candidate for a cost-effective neutron and plasma source for a component test facility (CTF) for fusion technology development. However, the disadvantage of compactness for a spherical tokamak reactor is that there is insufficient space for superconducting coils. The larger spherical tokamaks, MAST at Culham and NSTX at Princeton, are beginning to reach fusion-scale parameters. The stellarator is a fully three-dimensional toroidal configuration that requires no driven plasma current to confine the plasma—it is inherently steady state. But the three-dimensional coils are very hard to fabricate and the engineering for stellarator reactors remains challenging. A JET-scale stellarator is under construction in Germany.

## 7. Performance measures—what materials performance has been achieved?

The 14 MeV neutron flux across the walls of a reactor is expected to peak at about 2.5 MW m^−3^. This neutron bombardment will on average displace each atom in nearby parts of the blanket and supporting structures from its equilibrium position some 30 times a year. Displaced atoms normally return to their original configuration (when thermal vibrations bring displaced atoms together with vacancies). Sometimes, however, the vacancies and displaced atoms migrate differently, in which case accumulations at grain boundaries can produce swelling or embrittlement and weaken the material. Because they have much higher energy, fusion neutrons will initiate nuclear reactions that produce helium inside the structural materials about 100 times more copiously, per atomic displacement, than fission neutrons. There is serious concern that the helium could accumulate and further weaken the structure. Clearly, tests must be done. The neutrons also inevitably produce many other nuclear reactions in the structural material. By careful choice of elements it is possible, however, to ensure that essentially no long-lived radioactive isotopes are produced—such materials are termed low-activation materials.

It had been thought that only exotic materials (such as silicon carbide composite ceramics) could survive fusion neutron damage for long periods. The discovery during the 1990s, in tests at fission reactors, that special low-activation (body centred cubic) steels can probably survive in fusion reactor conditions for around 5 years before they would have to be replaced was, therefore, a very positive and welcome surprise. Silicon carbide composites that could operate at very high temperature (perhaps above 1000^°^C), and hence produce power with high thermodynamic efficiency, remain attractive in the long term.

The so-called plasma-facing materials and the exhaust structure called the divertor (through which particles, impurities and the helium ‘ash’ produced in D–T fusion are exhausted) will be subjected to fluxes of plasma particles and electromagnetic radiation. Typically, reactor designs require particle and radiation fluxes of 500 kW m^−2^ to the walls and 10 MW m^−2^ onto the divertor plates. The divertor and plasma-facing wall must resist erosion and survive many years in the hostile reactor environment. Special materials solutions are required and have been proposed for these areas, but they need further development and testing in reactor conditions (see below).

## 8. What remains to be done? The science and technology challenges for fusion power

A number of technical challenges must be met in order to progress to a demon- stration reactor and commercial fusion power. The critical goals are as follows.

— To sustain a high-gain burning plasma. This requires demonstrating that large amounts of fusion power (greater than 10 times the input power) can be produced in an essentially self-heated (by fusion alpha particles) plasma without provoking uncontrolled instabilities, over-heating the surrounding materials or compromising the purity of the fusion fuel. The plasma current must be driven so that steady state is also demonstrated. ITER (see below) is designed to attain this goal.
— To develop a reactor-compatible plasma exhaust system. This involves (i) optimizing a divertor magnetic field design that steers the particles away from the plasma and spreads the heat over the largest possible area; and (ii) developing appropriately tough materials for the reactor walls and divertor.
— To fabricate and test long-lasting, low-activation structural materials. This requires continued research and testing with reactor-level neutron fluences. The International Fusion Materials Irradiation Facility (IFMIF; see below) will provide the critical tests.
— To design and prototype fusion blankets. Blanket concepts have been developed but, because they have never been tested with 14 MeV neutron fluxes, which is a huge challenge. The first blanket tests will be performed in the later stages of ITER.
— To develop reactor-relevant tritium-handling systems. Tritium has been handled on JET in small quatities but there is no actual operational experience of extracting tritium from a blanket.
— To integrate components into a reliable and maintainable reactor design. Progress on the previous goals will inform the evolution from present conceptual designs to the engineering designs of the first reactors. Making a reliable reactor is possibly the greatest challenge.

Meeting the above goals would deliver a fusion reactor with adequate performance. However, performance would be enhanced and the cost of fusion-generated electricity reduced if a further series of goals were met. These include the following.

— The development of a reactor-compatible, high-confinement, high *β* magnetic configuration. This requires further suppression of turbulence and the extension of the stable operating regimes. It would allow a reactor at lower field strength and, therefore, cheaper and perhaps simpler coils. Spherical tokamaks may point the way.
— The fabrication of high-temperature structural and plasma-facing materials. This would allow higher operating temperatures of the walls and blanket structure, which would increase thermodynamic efficiency.

Recognizing the challenges outlined above, Culham developed in 2004 the ‘Fast Track to Fusion’—a strategic plan to develop fusion power (see below and UKAEA FUS 521 2005). This plan outlines the facilities that are needed and the expected timescales. We argue that this almost certainly *must* include a device to test the key engineering components—the CTF. Although this facility was proposed as a possible option in UKAEA FUS 521 (2005) we now consider it essential.

## 9. What next?

### (a) The final science stages—the International Tokamak Experimental Reactor and the International Fusion Materials Irradiation Facility

It is clear that much of what is required is technology rather than scientific development. Two intermediate facilities, ITER and IFMIF, are necessary to complete the science for the first generation of reactors. In the Fast Track they are to be constructed in 10 years, facilitating the start of construction of a demonstration reactor, DEMO, in 20 years. We provide only a short summary of ITER and IFMIF here—further details can be obtained from Ikeda (2007) and Moeslang *et al.* (2006).

#### (i) The International Tokamak Experimental Reactor

**Figure 4.**
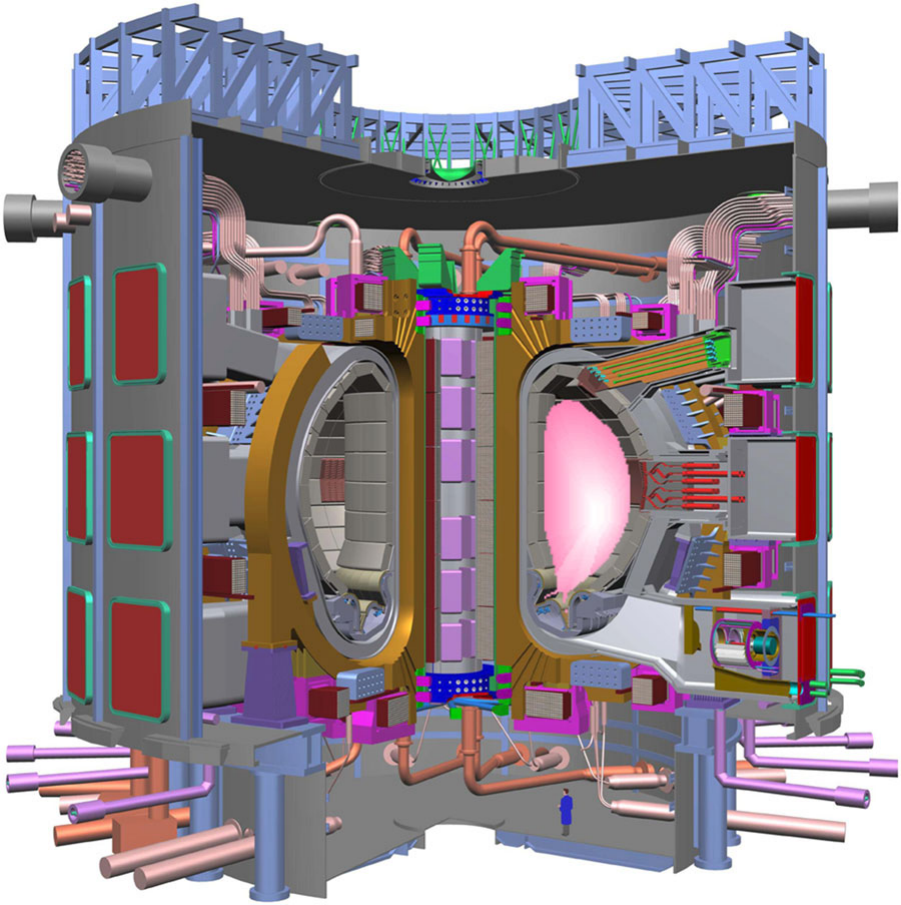
The ITER project, ready for construction, is designed to produce at least 500 MW of fusion power. It is similar in configuration to JET but twice as large (in each dimension) and it uses superconducting coils.

ITER, which is shown in figure 4, will be approximately twice the size of JET in linear dimensions, and operate with a higher magnetic field and current flowing through the plasma. The aim of ITER is to demonstrate integrated physics and engineering on the scale of a power station. The design goal is to produce at least 500 MW of fusion power, with an input of approximately 50 MW. The engineering systems of ITER have all been tested in smaller devices but not at this scale or all together.

The mission of ITER is complex and detailed but it can be summarized in three goals.

— Sustain a high-gain (*Q*>10) burning plasma. Recent studies indicate that the gain on ITER may exceed the *Q*=10 target and approach ignition (Budny 2009).
— Access high-performance steady-state regimes of tokamak operation. The aim is to extend high *β*, high-confinement (low turbulence), steady-state regimes found in smaller tokamaks.
— Test the first blanket modules at moderate neutron flux. ITER can deliver approximately 30 per cent of the expected neutron flux in a reactor—thus, low-power blanket tests can be performed. However, ITER will operate for at most a few hours per day; therefore, the total neutron fluence is not sufficient for a test of the blanket materials.

ITER is being funded and built by a consortium of the European Union, Japan, Russia, USA, China, South Korea and India. The design has recently been reviewed and updated, having been frozen since negotiation of the Agreement began in 2001, and the construction cost (originally estimated at €5 billion in 2008 prices) is currently under review. Prototypes of key ITER components have been fabricated by industry and tested. The site, at Cadarache in France, has been cleared and construction of components is beginning. The initial activity was slower than expected but the project is now gaining momentum.

#### (ii) The International Fusion Materials Irradiation Facility

Various materials are known that may be able to remain robust under neutron bombardment characteristic of a fusion reactor. It is, in any case, foreseen that the most strongly affected components will be replaced periodically. However, before a fusion reactor can be licensed and built, it will be necessary to test the materials for many years under power station conditions. The only way to produce neutrons at the same rate and with essentially the same distributions of energies and intensity as those that will be experienced in a fusion power station is by constructing an accelerator-based test facility known as IFMIF (International Fusion Materials Irradiation Facility). IFMIF will operate for 24 h a day, every day.

IFMIF, which will cost approximately €1B, will consist of two 5 MW accelerators that will accelerate deuterons to 40 MeV (very non-trivial devices). The two beams will hit a liquid lithium target that will produce neutrons, stripped out of the deuterons, with a spread of energies and an intensity close to that generated in a fusion reactor. These neutrons will provide estimated displacement rates (in steel) of 50, 20 and 1 displacements per atom per year over volumes of, respectively, 0.1, 0.5 and 6 l.

The priority at IFMIF will be to fully test the relatively conventional materials that are likely to be used in early fusion power plants, but it is very important also to push forward the development of advanced materials (such as Si–C composites) that would allow higher blanket temperatures and hence greater efficiency in generating electricity. The European Union and Japan are currently spending €150 M on final design and prototyping for IFMIF. However, there is no commitment to proceed to construction, and no site has been agreed.

## 10. What next?

### (a) The final technology stages—component test facility and DEMO

As any nuclear engineer knows there is much more to a working reactor and commercial power generation than a demonstration of scientific feasibility. Several components of the future fusion reactor—in particular, the systems that convert neutron power to electrical power—have yet to be tested at any scale. We believe that testing is essential and a CTF should be built (here we add an element to the usual fast-track scenario) to test wall and blanket components at full power. In our opinion a CTF will be needed before DEMO. But in any case it will be needed in parallel with and beyond DEMO to optimize components for succeeding reactors. The final technology integration must take place in a demonstration reactor, DEMO. The fast track calls for construction of DEMO (the first power plant, albeit an experimental one) to begin in 20 years.

#### (i) Component test facility

A CTF would be a compact, affordable (€1B), driven fusion device that can deliver reactor-level neutron and heat fluxes over many square metres. It would not be required to make net power. Instead, it would be dedicated to testing whole components of the blanket and wall at full power for many years. The materials supporting the blanket must retain structural integrity under these very challenging conditions. Several blanket designs will face their first nuclear tests in the later stages of ITER operation.

The spherical tokamak is the prime candidate for a CTF. Culham has pioneered spherical tokamaks and currently operates MAST (the MegaAmp Spherical Tokamak) that has achieved near fusion plasma conditions at very modest scale. The National Spherical Tokamak Experiment (NSTX) at Princeton in the USA also operates at about the MAST scale. Both Culham (Voss *et al*. 2008) and a team in the USA (Peng *et al.* 2005) have developed conceptual designs of CTFs based on spherical tokamaks. These designs are compact and affordable. A pragmatic approach to hasten fusion would be to build CTFs in parallel with ITER. A vigorous programme of wall and blanket development on these test facilities coupled with ITER’s programme could pave the way for the first demonstration reactors in the 2030s. There are no plans for a CTF in the current international programme. But there should be.

#### (ii) DEMO—first electricity

The most comprehensive, power plant conceptual study was completed in 2005, in the framework of the European Fusion Development Agreement (EFDA; EU Studies 2004). This study provided important results on the viability of fusion power, and inputs to the critical path analysis of fusion development described above. The study assumed that the first fusion power stations will be based on a conventional (ITER/JET-like) tokamak. Details of this study are given in appendix A. Recent studies in Japan (Tobita *et al*. 2006) (building on experience from Culham’s spherical tokamak MAST and Princeton’s NSTX) have shown the advantages of power plants with smaller aspect ratio, i.e. somewhere between the conventional and spherical tokamak. Thus, unless ITER produces major adverse surprises, it is likely that DEMO and the first commercial power plants will be similar to ITER perhaps with a smaller aspect ratio.

The programme outlined here (based on the Fast Track proposal) reflects an orderly, relatively low risk, approach. It could be speeded up if greater financial risks were taken, e.g. by starting DEMO construction before full power *in situ* tritium generation and recovery have been demonstrated in CTF. The risks could be reduced—and the timetable perhaps speeded up—by the parallel construction of multiple machines at each stage. In particular, with an Apollo project approach, it would be desirable to start building a low-performance DEMO *now*, in parallel to proceeding in an orderly way via ITER and IFMIF to a superior DEMO; the lessons from actually constructing a DEMO, and confronting the systems engineering issues involved in building a real power station, would be invaluable.

## 11. Concluding remarks—when will fusion be commercially viable?

Fusion power is still being developed, and will not be available as soon as we would like. Initial estimates of the scientific challenge of confining plasmas at over 100 million degrees Celsius were clearly overly optimistic. The cost and scale of the development were also underestimated—fusion cannot be achieved at small scale. However, as JET has shown, it is now possible to achieve fusion plasma conditions. Furthermore, it appears that it will be possible to build viable fusion power stations, and it looks as if the cost of fusion power will be reasonable. But time is needed to develop the technology in order to ensure that fusion power will be reliable and economical, and to test under power station conditions the materials that would be used in their construction. High availability is probably the greatest challenge that fusion will face in the future. If anything like 75 per cent is going to be reached relatively quickly, further development of fusion technology and a systems engineering approach (focused on buildability, reliability, operability and maintainability, building on experience from fission) will have to be adopted very soon.

Assuming no major surprises, an orderly fusion development programme—properly organized and funded—could lead to a prototype fusion power station putting electricity into the grid within 30 years, with commercial fusion power following some ten or more years later. Fusion could therefore play an important role in the energy mix in the second part of the century. The possible role of fusion in Europe from now to the end of this century was studied by the Netherlands Energy Institute in 1998 (Lako *et al*. 1998). Some of the assumptions in this study no longer look reasonable (e.g. that in 2100 the cost of oil would be $30 a barrel!), although others still look sensible (e.g. the assumed cost of fusion power). All such scenario modelling is of course subject to enormous uncertainties, and should be seen as an exploration of what might happen—not a prediction of what will happen. That said, the results are rather robust. If an unlimited coal supply is assumed, and no constraints are put on consumption, coal will be dominant. On the other hand, if atmospheric CO_2_ was assumed to be limited to below 600 ppm or a carbon tax of $30 per tonne or more was assumed, fusion was found to play an important role. Fission will also play a major role while the price of uranium remains reasonable and in the longer term fast breeder reactors become accepted. The fact is that it is incredibly hard to meet expected energy demand with constraints on carbon (either introduced by society, or due to the increasing scarcity of fossil fuels).

Success in developing fusion as an effective large-scale source of power on earth is not guaranteed. However, given the magnitude of the energy challenge, and the relatively small investment that is needed on the (approx. $5 trillion per annum) scale of the energy market, accelerated/fast track development of fusion is fully justified in view of its enormous potential. With so few other options available to provide the world’s power as the availability (and willingness to use) fossil fuels decreases, we cannot afford *not* to develop fusion power. Fortunately, it appears to be within our reach.

## Appendix A. Power plant studies

Four models (A–D) were studied in the European Fusion Development Agreement (EFDA) study as examples of a spectrum of possibilities. Systems codes were used to vary the designs, subject to assigned plasma physics and technology rules and limitations, in order to produce an economic optimum. The near-term models (A and B) are based on modest extrapolations of the relatively conservative design plasma performance of ITER. Models C and D assume progressive improvements in performance, especially in plasma shaping, stability and protection of the ‘divertor’, through which helium ‘ash’ and impurities will be exhausted. Likewise, while model A is based on a conservative choice of materials, models B–D would use increasingly advanced materials and operate at increasingly higher temperatures (which would improve the ‘thermodynamic efficiency’ with which they turn fusion power into electricity).

The power plant study shows that the cost of fusion-generated electricity decreases with the electrical power output (*P*_e_) approximately as *P*^−0.4^_e_. It was assumed that the maximum output acceptable to the grid would be 1.5 GW. Given the increase in temperature and hence thermodynamic efficiency, the size and gross fusion power needed to produce *P*_e_=1.5 GW decreases from model A (with fusion power 5.0 GW) to model D (fusion power 2.5 GW). The cost of fusion-generated electricity is dominated by the capital cost. It therefore depends very sensitively on (i) the cost of borrowing money or discount rate (*D*) and (ii) the availability of the plant (*A*), the dependence being approximately *D*^0.6^ (for *D* in the range 5–10%) *A*^−0.6^. The cost figures below assume 6 per cent for *D* (in real terms) and *A*=0.75.

The generating costs estimated in the European power plant study (EU Studies 2004) decreased from 9 Eurocents per kW h for an early model A to 5 Eurocents per kW h for an early model D (these costs would decrease as the technology matures). Even the model A result would be competitive with other generating costs if there was a significant carbon tax, which now effectively exists in Europe with the Emissions Trading Scheme. If acceptable and necessary, larger plants (with *P*_e_>1.5 GW) would be more cost effective, as discussed above.

These cost figures should not be taken too seriously in detail. The main point is that the order of magnitude is not unreasonable. The conclusion of the power plant study is that economically acceptable fusion power stations, with major safety and environmental advantages, seem to be accessible through ITER with material testing at IFMIF, and intensive development of fusion technologies.
